# Intestinal Adenocarcinoma Arising from a Mature Cystic Teratoma

**DOI:** 10.1155/2019/7894581

**Published:** 2019-11-18

**Authors:** King Man Wan, Forough Foroughi, Rajni Bansal, Martin K. Oehler

**Affiliations:** ^1^Department of Gynaecological Oncology, Royal Adelaide Hospital, Adelaide, South Australia, Australia; ^2^Department of Anatomical Pathology, Royal Darwin Hospital, Darwin, Northern Territory, Australia; ^3^Department of Obstetrics and Gynaecology, Alice Springs Hospital, Alice Springs, Northern Territory, Australia; ^4^Discipline of Obstetrics and Gynaecology, Adelaide Medical School, University of Adelaide, Adelaide, South Australia, Australia

## Abstract

Mature cystic teratomas are the most common ovarian germ cell tumour and account for 10–20% of all ovarian neoplasms. Malignant transformation of mature cystic teratomas is rare and has an incidence rate of less than 1%. The most common malignancy are squamous cell carcinomas. Here we present the case of an intestinal adenocarcinoma which is an exceedingly rare malignant entity arising within a mature cystic teratoma. Clinical presentation, imaging and histopathological diagnosis are discussed and previously presented cases in the literature reviewed.

## 1. Introduction

Mature cystic teratoma (MCT) of the ovary accounts for 10–20% of all ovarian neoplasms and is the most common ovarian germ cell tumour. Malignant transformation is rare and reported to occur in approximately 0.17–0.8% of cases [1, 2]. The most common malignancy are squamous cell carcinomas but basal cell carcinomas, sebaceous tumours, malignant melanomas, adenocarcinomas, sarcomas, and neuroectodermal tumours have also been reported [3].

## 2. Case Presentation

**Figure 1:**
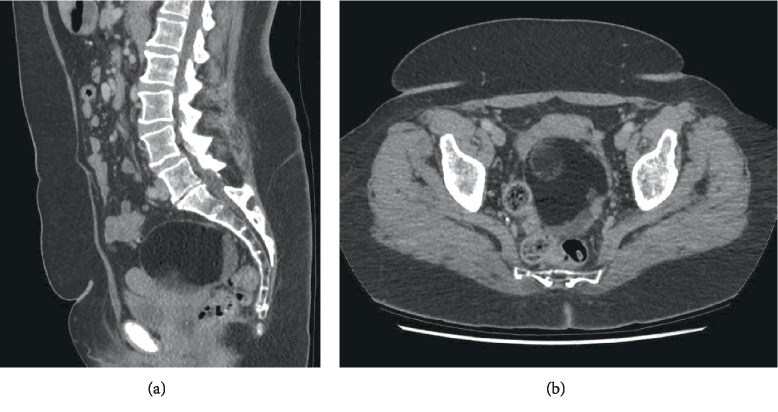
Preoperative CT images of the right adnexal mass.

A 58-year-old woman living in a remote region was referred to the local general gynaecological service for investigation of an episode of light postmenopausal bleeding. A pelvic ultrasound demonstrated a right sided complex adnexal mass measuring 101 × 70 × 89 mm and a borderline endometrial thickness of 5.1 mm. The mass had a well circumscribed outer capsule with no evidence of increased internal vascularity and there was not ascites. A CT of the abdomen and pelvis demonstrated the complex mass containing internal calcification, fluid, fat and soft tissue (Figure 1). There was no evidence of peritoneal or omental metastasis. The tumour markers showed an elevated CA 19-9 of 58 and normal CA 125 (14), CEA (2), AFP (4.1), HCG (<1) and LDH (220). The complete blood count as well as renal and liver functions were normal.

The patient's medical history was unremarkable and included one normal vaginal delivery. She had gone through menopause at age 48 and had not used any HRT. In her family history, she only had her sister with an early-stage endometrial adenocarcinoma.

The patient was referred to a tertiary gynaecological oncology service and after discussion at a multi-disciplinary team meeting with radiology review, the tentative diagnosis of mature teratoma with low risk for malignancy was made.

A hysteroscopy, dilatation and curettage of the uterus were performed and showed atrophic endometrium. The patient then underwent a total abdominal hysterectomy and bilateral salpingo-oophorectomy. Intraoperatively, she was found to have a mobile 10 cm left ovarian mass with no surface excrescences. The lesion was removed intact. The right adnexa, uterus and peritoneum were normal. Peritoneal washings were obtained. The operation was uncomplicated, and the patient was discharged from hospital on day 3 after the procedure.

Macroscopically, the left ovarian cyst measured 100 × 80 × 70 mm in diameter and had a maximum wall thickness of 2 mm. It contained yellow cheesy material, teeth and hair. The uterus, cervix and right ovary were unremarkable and histologically normal.

**Figure 2:**
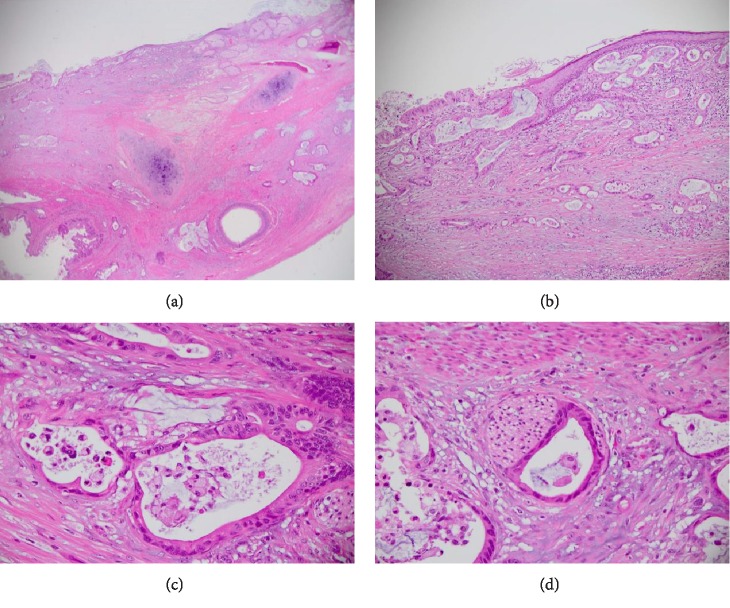
(a) Low power view of teratoma showing skin, glandular epithelium, and cartilage. There is infiltration of malignant glands within the cyst wall (H&E ×40). (b) Transition of benign squamous epithelium to dysplastic surface glandular epithelium and underlying invasive atypical glands with intraluminal dirty necrosis. (c) Invasive glands lined by intestinal-type epithelium and containing intraluminal necrosis. (d) Perineural invasion of infiltrating glands.

Histology of the ovarian cyst showed mature cystic teratoma, predominantly comprised of skin and adnexal elements with large areas of foreign-body type granulomatous response to hair. One section of the cyst was lined by dysplastic columnar type epithelium in continuity with squamous epithelium. Within this area, there were atypical irregular glands infiltrating the underlying stroma. The atypical glands were lined by pleomorphic cells with hyperchromatic enlarged nuclei and luminal dirty necrosis (Figures 2(a)–2(d)).

**Figure 3:**
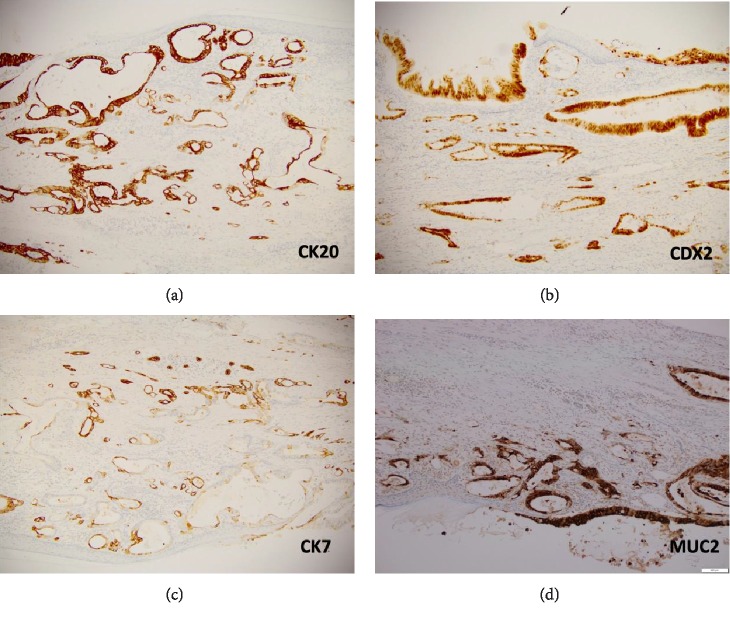
(a) CK20 is strongly positive in tumour. (b) CDX2 in strongly positive in tumour cell nuclei. (c) CK7 is patchy variable positive. (d) MUC-2 is strongly positive.

Immunohistochemical staining showed the tumour cells were strongly positive for CK20, CDX2, focally positive for CK7 and negative for CD30, PAX-8, Vimentin, ER, PR, and CD10 (Figures 3(a)–3(c)). MUC-2 staining was also performed and showed cytoplasmic positivity in tumoural cells consistent with intestinal differentiation (Figure 3(d)). Perineural invasion was identified, but no lympho-vascular invasion or surface involvement. No immature elements were found. The final diagnosis was intestinal-type moderately differentiated adenocarcinoma arising within a mature cystic teratoma.

The case was re-discussed at the multidisciplinary gynaecological oncology meeting and the disease staged as FIGO Stage 1A ovarian intestinal adenocarcinoma arising within a mature cystic teratoma. She was recommended to have adjuvant platinum-based chemotherapy to decrease risk of recurrence. However, after medical oncology review, the patient elected for observation only. She is alive and well after 12 months of follow up.

## 3. Discussion

MCTs or dermoid cysts are the most common ovarian germ cell tumour. They arise from totipotent cells in the ovary which develop into fully differentiated ectodermal, mesodermal, and endodermal tissue. Parthenogenetic activation of oocytes (embryonic development without a male gamete) is the most widely accepted theory for the origin of MCTs, primarily because of presence of 46, XX karyotype in almost all mature teratomas [4].

Intestinal adenocarcinomas arising within cystic teratomas are exceedingly rare and this is only the 12^th^ reported case in the literature (Table 1). Intestinal adenocarcinomas are suspected to arise from the endodermal cell line with prevailed derivation of the lower gastrointestinal tract structure, thereby demonstrating characteristics of intestinal differentiation in immunohistochemistry, with CK20 and CDX2 positivity [5, 6]. Focal positivity for CK7 in our case was misleading initially as it suggests a primary ovarian mucinous tumour. However, subsequent positive staining with MUC2 confirmed the diagnosis.

The mechanisms of malignant transformation in ovarian MCTs are uncertain. MCTs are thought to result from replication errors during meiosis and they may represent primary oocytes that have escaped from meiotic arrest. However, it is unclear how subsequent malignant transformation occurs. A systematic genomic evaluation of ovarian SCC arising in MCT showed similarities to other non-HPV SCC, but with distinct features, including bi-allelic TP53 mutations [7]. Further research will be required to address the question of MCT cell of origin and to understand what causes transformation of the MCT into malignancies.

It has been suggested that prolonged exposure of MCTs to carcinogens in the pelvic cavity might promote malignant transformation, as MCTs are usually detected 15–20 years earlier than malignant transformations [8]. If this might warrant a more pro-active removal of MCTs to avoid long term malignant transformation is unknown.

Predictive factors for malignant transformation of MCTs include old age, large tumour size, raised CA125, postmenopausal status and presence of solid components [6]. However, 80% of malignant transformations have been reported in women of reproductive age [7]. Ultrasound imaging may show branching isoechoic components and magnetic resonance imaging especially fat-suppression images, may increase preoperative suspicion for malignant transformation [9, 10]. Tumour markers are inconsistently reported but elevated CEA and CA19.9 may indicate malignant transformation. Elevated serum squamous cell carcinoma (SCC) antigen levels can help differentiating between benign and MCTs with malignant transformation, but is probably not helpful in intestinal adenocarcinomas and rarely used in routine clinical practice [11].

Diagnosis is ultimately made after surgical resection and histopathological analysis. Immunohistochemistry with CDX2, CK20 and CK7 helps to differentiate between colorectal adenocarcinomas and extra-intestinal gastrointestinal adenocarcinomas [12]. However, histopathology cannot differentiate in between primary intestinal adenocarcinoma and metastatic deposits from a primary gastrointestinal cancer and therefore gastrointestinal tract assessment should be performed.

The mainstay of treatment is surgical resection and complete staging. In most cases the malignancy is confined to the ovary and care should be taken to prevent rupture of MCTs with potential tumour spillage. Optimal surgical cytoreduction may be beneficial for metastatic disease [13]. Cases with disease confined to the ovary are usually managed by observation alone. In advanced stage or cases with tumour spillage, adjuvant therapy has been given. However, no standardised chemotherapy exists due to its rarity and regimes include platinum- and 5FU-based treatments [14]. KRAS mutations have been identified in some of these cancers which indicates that targeted therapies against the epidermal growth factor receptor could be effective [14]. Limited evidence exists for the use of chemoradiation [2].

Poor prognostic factors for malignant transformation of MCTs include tumour dissemination, cyst wall invasion, ascites, spontaneous or accidental rupture, adhesion, and some tumour types other than squamous carcinomas [15].

Prognosis of MCTs with malignant transformation has traditionally been reported to be poor as a large number of women die within the first year after diagnosis. However, the majority of these women had MCTs with squamous cell carcinomas at various stages. Intestinal adenocarcinomas in MCTs may have better prognosis and 9 of the 10 reported cases in the literature were diagnosed at FIGO Stage 1. Eight of those cases did not receive adjuvant therapy. Recurrence rates of intestinal adenocarcinomas in MCTs are unknown, but long-term survival and probable cure was reported for women with Stage 1A disease [16]. However, metastatic disease is associated with poor prognosis and one patient who suffered from Stage IIIC disease died 3 months after surgery.

## 4. Conclusions

Intestinal-type adenocarcinoma arising in MCTs are very rare malignancies and preoperative diagnosis is difficult. Histopathological diagnosis is aided by the use of the immunohistochemical markers, CK 7, CK20 and MUC2. Most cases are diagnosed at Stage 1 and tend to have a better prognosis than other forms of malignant transformation.

## Figures and Tables

**Table 1:** Reported cases of intestinal adenocarcinoma arising from a mature cystic teratoma (TAH: total abdominal hysterectomy, BSO: bilateral salpingo-oophorectomy, OM: omentectomy, APT: appendectomy, PALN: Para-aortic lymphadenectomy, 5-FU: 5-fluorouracil, FAMT: 5-fluorouracil + endoxan + mitomycin-C + toyomycin, P + C: paclitaxel + carboplatin. NR: not reported).

Case	Author	Age	Ca 19-9 (U/ml)	Tumour size	Surgery	FIGO stage	Adjuvant therapy	Follow up
1	Fishman A (1998)	38	NR	20 × 13 × 8.5 cm	TAHBSO + OM + appendix	IIIC	5-FU + leucovorin	Died 3 months after surgery
2	Ueda G (2003)	62	NR	35 cm	TAHBSO	IA	5-FU, mitomycin C, toyomycin cyclophosphamide	15 years
3	Kushima M (2004)	52	109	6.4 × 4.8 × 2.8 cm	BSO	IA	None	31 months
4	Levine DA (2004)	37	NR	15 × 12 × 11 cm	USO + OM + pelvic and PALN	IA	None	40 months
5	Guney M (2006)	38	>1000	NR	TAHBSO + OM + pelvic and PALN	IA	None	NR
6	Min KJ (2006)	77	NR	17 × 14 × 2 cm	TAHBSO	IA	None	12 months
7	Takai M (2012)	49	3.8 (normal)	6.7 × 5.7 cm	TAHBSO + OM	IA	NR	5 years
8	Hershkovitz D (2013)	13	162	7 × 10 cm	NR	IA	NR	5 months
9	Li Y (2014)	51	41.9	5.8 × 4.5 cm	TAHBSO + OM + appendix	IA	Single IP carboplatin + IV carboplatin + paclitaxel	13 months
10	Li Y (2014)	43	>1200	10.8 × 9.7 cm	TAHBSO	IA	None	11 months
11	Clark M (2016)	42	349	17 × 12 × 7.5 cm	TAHBSO + OM + node sampling	IA	None	12 months
12	Wan K (Current)	58	58	10 × 8 × 7 cm	TAHBSO	IA	None	12 months
